# Caregiver’s burden at the end of life of their loved one: insights from a longitudinal qualitative study among working family caregivers

**DOI:** 10.1186/s12904-022-01031-1

**Published:** 2022-08-10

**Authors:** Femmy M. Bijnsdorp, Bregje D. Onwuteaka-Philipsen, Cécile R. L. Boot, Allard J. van der Beek, H. Roeline W. Pasman

**Affiliations:** 1 Department of Public and Occupational Health, Amsterdam UMC, Vrije Universiteit Amsterdam, Amsterdam Public Health Research Institute, Expertise Center for Palliative Care, Amsterdam, Netherlands; 2 Department of Public and Occupational Health, Amsterdam UMC, Vrije Universiteit Amsterdam, Amsterdam Public Health Research Institute, Amsterdam, the Netherlands

**Keywords:** Family care, Paid work, Burden, Life-threatening illness, End-of-life, Interview study, Longitudinal

## Abstract

**Background:**

Growing numbers of people with advanced illnesses who wish to die at home, a concurrent decline in the accessibility of professional home care, and policies aiming at prolonging work participation are increasing the reliance on family caregivers. This study aimed to describe trajectories in burden of working family caregivers who care for patients with a life-threatening illness, and identify factors in work and care that are related to changes in burden over time.

**Methods:**

Semi-structured interviews were held in one to four rounds between July 2018 and November 2020 with 17 working family caregivers of patients with a life-threatening illness living at home. Transcripts were analysed as a single unit to create timelines per participant. Next, individual burden trajectories were created and grouped based on the course of burden over time. Factors related to changes in burden were analysed, as well as similarities and differences between the groups.

**Results:**

It was common for family caregivers who combine work and end-of-life care to experience a burden. Two trajectories of caregiver burden were identified; caregivers with a persistent level of burden and caregivers with an increasing burden over time. Family caregivers with a persistent level of burden seemed to be at risk for burnout throughout the illness trajectory, but were often able to cope with the situation by making arrangements in care or work. Caregivers with an increasing burden were unable to make sufficient adjustments, which often resulted in burnout symptoms and sick leave. In both groups, burden was mostly related to aspects of the care situation. The emotional burden, a decreasing burden after death and a different view o﻿n the trajectory in hindsight proved to be important overarching themes.

**Conclusions:**

Providing care to a loved one nearing the end of life is often emotionally burdensome and intensive. To facilitate the combination of paid work and family care, and reduce the risk of burnout, more support is needed from employers and healthcare professionals during the illness trajectory and after death. Bereaved family caregivers also warrant more attention from their supervisors and occupational physicians in order to facilitate their return to work.

**Supplementary Information:**

The online version contains supplementary material available at 10.1186/s12904-022-01031-1.

## Introduction

Due to the growing number of people with advanced illnesses who wish to die at home and a concurrent decline in the accessibility of professional home care, there is an increasing reliance on family caregivers to care for patients with a life-threatening illness at home [1]. Care demands largely depend on the type of illness of the patient. In general, three distinct disease trajectories until death have been described: acute decline in functioning (e.g. aggressive cancers), fluctuating deterioration (e.g. organ failure) and gradual decline (e.g. frailty or dementia) [2]. Each illness trajectory differs in the course of the decline in functioning, which implies different requirements in terms of the nature and intensity of care. Generally, when health deteriorates, the need for intensive care from professional caregivers as well as family caregivers increases [3].

In addition to the patient’s illness trajectory, previous research has studied trajectories in well-being and distress among family caregivers. A study using serial qualitative interviews has indicated that family caregivers of lung cancer patients shared much of the illness experience of the patient from diagnosis until death. Experienced distress in social, psychological and spiritual domains followed a similar pattern to the distress suffered by patients, and was mainly shaped by the nature of the illness and the availability of care for the patient [4]. Also, research has shown that about one in five family caregivers of patients at the end of life experienced a heavy care-related burden [5]. A recent systematic review has found that the most important factors related to burden in family caregivers were the duration of family care and the patient’s dependency level [6].

In addition to the illness trajectory and care situation, family caregivers’ experiences may be shaped by other factors as well. More than half of the family caregivers of patients at the end of life have paid work [5]. Analysis of first-round interview data from the current study has identified four domains that influenced the experiences and needs of working family caregivers of patients with a life-threatening illness. These domains were: family caregiver characteristics, the care situation, the situation at work, and the context (e.g. ability to share care, and communication with healthcare professionals, organizations or municipalities). In turn, their experiences and needs sometimes affected caregiver health and well-being, or prompted certain actions or strategies [7].

Previous research has shown that family caregivers in more demanding care situations are more likely to make changes to their work situation [8]. Another study has found that about a quarter of the caregivers of patients with advanced cancer had changed their work situation (e.g. reduced working hours, or taken leave) following the patient’s diagnosis. This change in work situation was associated with worse mental health [9]. However, the causal direction of the relationship between mental health and work changes remained unclear. Also, the aforementioned changes in the work situation could involve financial costs for both the caregiver and the employer. Caregivers might experience immediate losses in income and long-term losses in future retirement benefits if they are unable to reconcile work and care demands and/or either leave the workforce entirely or reduce working hours. Employers might experience costs related to replacing workers who leave the company, work interruptions, or productivity loss (e.g. absenteeism and/or presenteeism) [10].

Little is known about the various trajectories of caregiver burden related to combining paid work and family care at the end of life. This suggests that more attention is needed for the impact of both the characteristics of the care situation (e.g. disease prognosis, patient’s dependency level) and characteristics of the workplace on changes in caregiver well-being over time. Equally, more attention needs to be paid to the influence of caregiver burden on the provision of family care at the end of life and participation in the labour force over time. In addition, most studies regarding the combination of paid work and family care at the end of life are cross-sectional [11–14]. Prior longitudinal studies were quantitative, and a qualitative description of the experiences over time of working family caregivers at the end of life is lacking. The current study elaborates on an earlier cross-sectional study [7] by performing longitudinal qualitative analyses using several rounds of in-depth interviews with working family caregivers of patients with a life-threatening illness. The aim was to describe the trajectories in the burden for working family caregivers of patients with a life-threatening illness, and to determine which factors in paid work (e.g. demands and resources) and care (e.g. intensity and illness progression) are related to changes in burden over time. Knowledge about these trajectories can help family caregivers, employers and healthcare professionals to obtain or provide timely and sufficient support in order to reduce or prevent undesirable consequences of combining family care and paid work.

## Methods

### Design and participants

This study was designed as a longitudinal qualitative study using in-depth interviews with working family caregivers of patients with a life-threatening illness. Family caregivers were recruited via general practitioners based in various regions in the Netherlands. Purposive sampling was used, ensuring as much variation as possible with regard to gender, working hours, sector, illness type, and intensity of care. The general practitioners handed out participant information letters to family caregivers of patients living at home with a life-threatening illness (e.g. incurable cancer, chronic lung disease, heart failure, dementia, or progressive neurodegenerative disorder), where the caregiver combined this task with paid work for at least twelve hours per week. In addition, family caregivers were recruited using convenience sampling via posters in several departments of a Dutch academic hospital and an item in the hospital’s corporate newsletter. Interested family caregivers could contact the primary researcher (FB) and were provided with the participant information letter.

An online questionnaire was completed by participating family caregivers, providing information about their gender, age, education, work characteristics (e.g. employed or self-employed, working hours per week and sector), and characteristics of the care situation (e.g. relationship, illness type, caregiving tasks, intensity of care, and place of residence of care recipient). Participants were included if they were aged 18 or older, provided family care for at least one hour per week to a patient with a life-threatening illness who lived at home, and combined this with paid work for at least twelve hours per week, all at the start of the study. Participant characteristics are presented in Table 1. The consolidated criteria guidelines for reporting qualitative research (COREQ) were followed for reporting on qualitative data (see Additional file 1) [15]. In addition, the criteria for methodological rigor in qualitative studies adapted from Lincoln and Guba were followed (see Additional file 2) [16, 17].

**Table 1:** Characteristics of participants according to the trajectories of burden (*n* = 17)

**#**	**Number of interviews**	**Gender**	**Age**	**Education**	**Employment**	**Sector**	**Work **^**a**^	**Care **^**a**^	**Care recipient**	**Type of illness**	**Contact frequency**	**(One of) Care recipient(s) died during study**
**Trajectory: Persistent moderate level of burden**												
3	3	Female	40–45	Middle	Employed	Health and social care	36	5	Parents (in-law)	Cancer / dementia	Daily	Yes
11	3	Male	65–70	High	Employed &	Business + FMCG	12	28	Partner /	Stroke / organ failure	Lives in same house	No
					Self-employed				Parent (in-law)			
14	3	Female	35–40	High	Employed	Health and social care	24	50	Child	Cancer	Lives in same house	No
16	3	Female	50–55	Middle	Employed	Health and social care	20	10	Parents (in-law)	Progressive neurodegenerative disorder / other	Daily	Yes
**Trajectory: Persistent high level of burden **^**d**^												
1	3	Female	50–55	Middle	Employed	Health and social care	36	15	Parents (in-law)	Dementia / cancer, other ^b^	Weekly	No
4	3	Female	50–55	Middle	Employed	Health and social care	24	25	Partner	Progressive neurodegenerative disorder	Lives in same house	No
5	3	Female	55–60	High	Employed	Business	39	14	Parents (in-law)	Dementia, stroke, other / organ failure, other	Daily	No
7	4	Female	60–65	Middle	Employed	Public services	18	90	Partner	Progressive neurodegenerative disorder; organ failure	Lives in same house	No
8	4	Female	55–60	Middle	Employed	Health and social care	32	8	Parent (in-law)	Dementia; cancer	Daily	Yes
13	3	Female	55–60	High	Employed	Education	24	10	Parent (in-law) / other family member	Cancer / dementia	Weekly	No
17	4	Male	30–35	High	Employed	Public services	36	12	Parent (in-law)	Cancer	Daily	Yes
**Trajectory: Increasing level of burden **^**d**^												
2	3	Female	35–40	Middle	Employed	Health and social care	16	12	Partner	Cancer	Lives in same house	Yes
6	3	Male	50–55	Middle	Employed	Health and social care	20	30	Partner	Organ failure; dementia; other	Lives in same house	No
10	3	Female	40–45	Low	Self-employed	Creative arts	40	15	Parents (in-law)	Dementia / stroke	Daily	No
12	4	Male	40–45	High	Employed	Education	40	16	Parent (in-law)	Cancer	Daily	No
15	3	Female	50–55	High	Employed	Public services	28	25	Partner	Progressive neurodegenerative disorder	Lives in same house	Yes
18	1 ^c^	Male	30–35	High	Employed	Public services	36	15	Partner	Cancer	Lives in same house	Yes

^a^in hours per week; ^b^other included: rheumatism, psychiatric disorder, frailty, disability; ^c^relative died shortly after application to study, therefore only interviewed once; ^d^Family caregiver #4 and #18 had slightly different characteristics in the trajectory

### Data collection

Semi-structured interviews were held in one to four rounds between July 2018 and November 2020. Interviews were held every six months or at a different frequency if indicated by the disease (e.g. one-month intervals in caregivers of advanced cancer patients, but eight- to ten month-intervals in caregivers of people with dementia or organ failure, which progressed less rapidly) [18]. Eighteen family caregivers participated in the first round of interviews, in which data was collected until no new themes emerged. The participating family caregivers were then followed for a longer period of time in order to describe their trajectories in burden. One participant withdrew from the study after the first interview for unknown reasons and was not included in the current analysis. The relative of one of the other participants died shortly after the caregiver had agreed to participate. This family caregiver was therefore only interviewed once. However, the caregiver’s experiences with the situation and timeline of care and paid work were discussed in retrospect, and therefore this participant was included in the analysis. In total, 17 participants were included for analysis in the current study.

All interviews were conducted by one female researcher who was trained in in-depth interviewing (FB). The interviews were guided by a topic list, which addressed the personal situation (e.g. own health, care situation and work situation), experiences with the combination of work and care, and support and other needs. Communication (e.g. at work, with healthcare professionals, with organizations or municipalities, or with the care recipient or family) was added to the topic list after analysing the first-round interviews as this proved to be an important topic for family caregivers [7]. In subsequent rounds of interviews, special attention was paid to changes in these topics over time and previous interviews were reread in preparation. At the end of each interview, the most important themes and changes were summarized by the interviewer and checked by the participant. The interviews were held at the family caregiver’s own home, at the premises of VU University Amsterdam, or via video calling. All interviews were audio-recorded and the duration varied between 45 and 120 min with a total of almost 64 h of recordings. Ad hoc field notes and a summary of the conversation were noted down after each interview, which provided input for the subsequent interviews.

### Data analysis

**Fig. 1:**
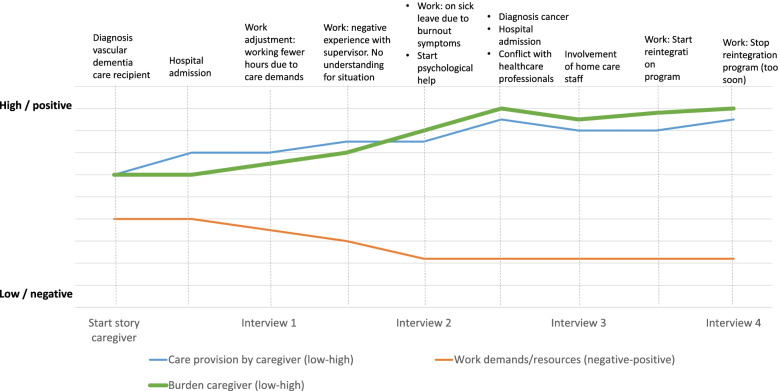
An example of an individual graph of developments in burden, work and care. Note: This is an example of the development in burden, family care provision and work demands/resources over time. This example was created based on the experiences of multiple participants to ensure privacy, since the trajectory of one person including all specific events in this trajectory contains identifiable information. The changes in the lines are based on statements of the family caregiver and do not represent absolute scores

The interviews were transcribed verbatim and were analysed in ATLAS.ti 9 following the principles of thematic analysis [19, 20]. The themes and framework that arose from the first round of interviews have been described in more detail in the first paper of this study [7]. The longitudinal analysis used in the current paper was based on 53 interviews, carried out with 17 participants over a time period of 2.5 years. All transcripts from each participant were analysed as a single unit to investigate individual experiences and changes over time. Individual trajectories were then drawn in a graph with separate lines indicating changes over time in the care intensity (low–high), work demands/resources (negative–positive) and caregiver burden (low–high) to grasp the large amount of data. This provided a first overview of the individual trajectories (including changes in burden, work and care) of the family caregivers (see Fig. 1 for an example). In each interview, family caregivers indicated whether and how the care situation had changed compared to the previous interview, and if the care had become less or more intensive (in hours or in their perception). The work line indicated the balance between demands and resources at work, and was based on changes in experienced support at work, flexibility, work adjustments or work pressure. The level of caregiver burden was based on statements that the situation was more or less demanding, exhausting or burdensome compared to the previous interview, and whether caregivers spoke of strain, distress, crossing their own boundaries or the fact that the situation was starting to take its toll. Within the individual trajectories, specific events related to the aforementioned topics (e.g. hospital admissions, transition to nursing home, or start of new job) were marked in the timelines to gain insight into factors that triggered changes. The trajectories of five participants were analysed and discussed by FB, HRP and BP. Discrepancies in the trajectories made by the three researchers were discussed until consensus was reached. The remaining trajectories were analysed by FB. After that, the trajectories were grouped based on the burden line, to create groups with a similar care burden course over time. Factors related to changes in burden were analysed, as were similarities and differences between the resulting groups. The groups were extensively discussed by all team members throughout the analytic process.

## Results

Qualitative analysis revealed that all family caregivers experienced a burden to some extent over the course of the illness trajectory. Broadly, two groups were identified, each with a similar course in the level of caregiver burden over time. The first group had a persistent (moderate or high) level of caregiver burden, and the second group experienced increasing caregiver burden over time. In both groups, caregiver burden was mostly related to the care situation (e.g. intensity, dependency of the care recipient, illness progression and acute care situations). In some cases, the work situation (e.g. irregular working hours, little support and understanding from supervisors, and/or mental workload) also caused a heavy burden.

 The group that experienced a persistent level of burden differed from the group with an increasing burden. The caregivers with a persistent level of burden indicated that they were at risk of burnout throughout the trajectory, but somehow managed to cope with the situation and remained active at work. However, the caregivers with an increasing burden experienced more and more burnout symptoms over time and were on sick leave from work at some point. Caregivers with a persistent level of burden were often able to make adjustments to the situation, using support resources in care and/or paid work, especially at times that required intensive family care.

The trajectories of caregiver burden and their relation to developments in paid work and family care are described in more detail below. This is followed by a discussion of overarching themes that proved to be important for both trajectories. These themes related to the emotional burden of caring for someone nearing the end of life, decreasing burden after death, grief and the return to work, and having a different view on the trajectory in hindsight.

### Persistent level of burden

In the first group, family caregivers experienced a persistent (moderate or high) level of caregiver burden, despite some small fluctuations, over the course of the illness trajectory. These family caregivers often provided care to more than one person or intensive care to a partner. The balance between resources and demands at work was generally stable and positive over time. The caregivers were able to make use of flexibility, working remotely and autonomy at work, but the availability of such options was often not directly linked to fluctuations in the level of burden. Temporary increases in burden were related to transitions in care (e.g. when the care recipient moved to a nursing home), hospital admissions and returning home after a hospital stay (Table 2, Q1), periods of intensive caregiving, deteriorating health of the care recipient, or death of the care recipient. Pressures when arranging care and poor communication with healthcare professionals, municipalities and other organizations were sometimes also associated with a higher burden. Family caregivers who experienced a persistently high burden over time mentioned that their own capacity decreased over time and that their stress levels were constantly high (Table 2, Q2). Often, the burden stabilized (temporarily) or even decreased slightly when family caregivers could share care tasks with others, when the intensity of care decreased or stopped, when the care recipient moved to a hospice (Table 2, Q3), or when work adjustments were made (e.g. working fewer hours). Sometimes psychological help to cope with the situation or a coaching programme also stabilized or decreased the burden. Although the family caregivers in this group generally had resources at work and help from others, the level of burden remained relatively high over time. In one case, the burden was at a persistent level, but decreased substantially after the caregiver changed jobs (#4, Table 1). The new job was easier to combine with family care (e.g. no weekend or night shifts, less complex tasks) and the new supervisor was more supportive. Family caregivers noted that providing intensive care and/or care to multiple care recipients was very exhausting, and caregivers reported that their mental fatigue increased over the course of the illness trajectory (Table 2, Q4).

**Table 2:** Quotes trajectories

No	Trajectory	Theme	Quote
Q1	Persistent high level of burden	Returning home after hospital admission	I think in particular it’s the times when… well, my mother got admitted to hospital a couple of times and then she came home again. Because those are the moments when I don’t know how she is — what can she do, what can’t she do? And then I have this very strong feeling that it’s really not quite on, but she is still coming home so I have to sort stuff out if it goes wrong. Or now I have to make sure it goes well. And those were moments that caused an awful lot of stress. You know, that I don’t really know what’s going to happen and whether it’ll work out or not. (#17, T3)
Q2	Persistent high level of burden	Own capacity reduced	Yes, tough. In a word: tough. […] Well, I’m finding now that I’m getting physical problems too. […] Right, it’s that short fuse situation; just one little thing has to go wrong and you are immediately in total stress mode. It could be your work, it could be something at home or having to deal with the authorities. […] And you know, it just gets worse and worse. I think I was better able to laugh things off in the past. I still try that now, but well… it’s a combination of getting older yourself, the care getting tougher and you’ve still got your work; you can’t always do the things you want to. Well, there are no specific moments, it can just happen. You know, things can suddenly boil over because stress levels are constantly so high. (#7, T3)
Q3	Persistent high level of burden	Burden decreases when care recipient moves to hospice	That was tricky. In that respect, a weight was lifted from my shoulders because I no longer had that care task, I didn’t have to be afraid all the time that I should be there or that something might go wrong. So that really lifted a weight off my shoulders. But I reckon I was probably round there more than before, but with a completely different attitude, more because I wanted to be there than because I had to be there. So it made a really big difference mentally. (#17, T4)
Q4	Persistent moderate level of burden	Increasing fatigue over the course of the illness trajectory	I was really tired mentally, because you’re still thinking about it, how it will progress, what will happen. And he left so suddenly [for the nursing home]. Well, then it just got… right, in the sense of tiring: you’re at work all day, you wait until the traffic has died down, then you drive to [the nursing home] and the next day you’re back with her [other care recipient]. I did find that physically tiring because I’m not used to having things on in the evenings as well. But I mainly found that it got harder and harder mentally. (#3, T3)
Q5	Persistent high level of burden	High expectations for themselves	Well, I still want to do it, whatever the cost. So, how can I explain it? You want to do it all… take my mother-in-law; she recently moved to T. and no one expects me to take on that task on top of everything else, because it all takes more time. In the past we did that… in the past, yeah, just six weeks ago… we combined it with my parents, dropping in on Mother, giving her medicines, food, and then back to see the others. Now it’s basically two hours extra and you can’t fit that in between other tasks. So nobody expects it, but I expect it of myself. Like I think I really should just pop along. Then that has to be on a particular Friday afternoon, so I’m working hard on planning things so that I can still go there, whatever it takes. So you really do it to yourself. I know I’m not the only one who’s like that. So yes, I’m actually totally opposed to family caregiving. (Laughs.) Well, opposed to the burden of family caregiving. (#5, T1)
Q6	Persistent high level of burden	Burden related to the death of one care recipient while caring for other care recipient and experiencing high work pressure	I’ve had a turbulent six months. So much has happened of course, settling everything after my father died, at any rate. Then the solicitors, the tax aspects, that kind of thing. So it was an awful lot of paperwork on top of the care for my mother. Well, this became quite a problem for me at the end of the year because I got given a new project through my work while I was still busy with other things. And then my head started to spin a lot and I realized that I was losing control, I didn’t have things properly organized and I was forgetting things. Then I thought to myself: you know what, I haven’t even been through my grieving process. Because I was immediately busy with my mother who is also still seriously ill. Then I realized that I simply didn’t have time for my own stuff. My head was so full that I kind of felt, well, I don’t know, if I read something I thought I’d never seen it before whereas in fact I’d read it a week earlier. So I really was a bit in the danger zone in terms of burnout or becoming overstressed. (#13, T2)
Q7	Persistent moderate level of burden	Critical about role of healthcare professionals	We’re now in a transitional phase with him for the oncological centre, which is centrally organized in U. in the Netherlands, so we had four different hospitals in the space of six months, all with different protocols. So you end up in the same treatment process every time, but everyone does it in a slightly different way. That means every day I pick out mistakes in the admission, in my child’s treatment, and I give them feedback about that too. I find that costs me an awful lot of energy because I’m always on the lookout — what’s happening, what are you giving him, why are you giving this, shouldn’t you have done that instead, could you please check? Then people say sure, but you also need to be the mother. That’s all very well but if you guys did your job properly then I’d be able to take a step back, but as long as I’m still picking out mistakes I’m not going to do that. I am the mother but I’m also the nurse, someone with expertise in that area. And that’s one area where I would much rather that the healthcare system had been a bit different. (#14, T1)
Q8	Increasing level of burden	Last months of care were most hard-going	The last few months. He couldn’t eat anymore, he couldn’t do anything anymore. All he could do was move his eyes. So that was basically the care. He had to be washed, the neighbourhood care service helped with that but I still did a lot myself. And the stoma had to be taken care of, so pretty much everything. He simply couldn’t do anything anymore. If something happened in the night, then I’d have to get up, and if the Tobi stopped working all he could do was make a sound — he couldn’t call me anymore. So I slept so lightly that I could even hear that. Yeah, that was basically a question of surviving. I really couldn’t cope anymore, I was exhausted. (#15, T3)
Q9	Increasing level of burden	Struggling to accept help	Well, they [the neighbourhood care service] had actually been saying that all along because of course they had noticed this already, before I had myself, but I always had this idea, you know, I always had this feeling that it would be a failure for me if I was to say I needed to let someone else take over. I just found it incredibly difficult personally. Even though I did notice that it was beginning to get more than I could cope with. So then they said a couple of times that they could take over some tasks from me if I needed, such as the washing. So there came a point in February, just after he got back from hospital, when I said I couldn’t cope. I can’t cope anymore. I’d wash him on Tuesdays and Thursdays, but I’d get home from work at two thirty, do the grocery shopping at three, cook, eat, take a shower, then I’d have to give him a shower and when I’d done all that I could go to bed, because I was finished. The day had ended. I thought I can’t keep that up anymore, because I was utterly worn out. So at some point in February, I said to the neighbourhood care service, right, I’m going to hand things over. And they did a perfectly good job. (#15, T2)
Q10	Increasing level of burden	Burnout symptoms	It’s kind of like excess adrenaline in your body. When you wake up, you’re immediately in this mode of how awful the situation is. As if you’re about to take a really important exam. So first I started taking quarters of lorazepam, but the GP said I should just take a half. So that’s going better. I take half a pill when I wake up and then that feeling goes away. The mornings are really the worst period to get through because you need to get used to what the situation is like again, then it goes better in the afternoons… So, I’m sleeping a bit less at night so I have a nap in the afternoons and that’s basically how I get through the day. (#2, T2)
Q11	Increasing level of burden	Burnout and PTSD from care situation	I’ve got a serious burnout, diagnosed PTSD, due to all my wife’s health problems and I’ve been off work for at least nine months. […] Of course this had been going on for a while but you hide it and you become skilled in the art of survival. But in the end, late August, I couldn’t cope anymore. The morning it happened, my moped wouldn’t start and then I’d had enough. I genuinely felt then that I couldn’t cope anymore – I couldn’t deal with it any longer, I wasn’t up to the tasks anymore. I was exhausted. There was so much stress. And yeah, no longer being able to continue. (#6, T2)
Q12	Increasing level of burden	Lack of sleep	The nights are interrupted because she needs help going to the toilet and then getting back into bed. Or she falls out of her bed. That happens a lot at the moment. We sleep separately because she can’t get up the stairs anymore. So she actually has to give me a call, then I come downstairs… but she also gets panic attacks at night or epileptic fits. (#6, T2)
Q13	Increasing level of burden	Unsupportive reaction after calling in sick	Right, I find it incredible. We work for the health service, so we say we believe people are important and we want to care for them. Only that’s not true when it’s us as people. And not at all when it’s for a colleague who’s already in a difficult situation. When they report sick, just accept it, say you’ll pass on the message and get better soon, or something like that. But don’t start talking about all the problems it will cause and how full the schedule is, that all kinds of arrangements will have to be made and that this really isn’t a good time. Someone literally said that to me… And I really didn’t like that at all. (#6, T2)
Q14	Increasing level of burden	Negative experience with supervisor/manager	I asked to have a chat and I explained that I was having to do more and more caregiving. That my wife wanted me to give the care. And that I would simply need to cut my working hours. […] My manager’s first response wasn’t that positive, not saying OK, we’ll do that. He said, well, I’ll need to see whether I’m still getting anything out of you. […] So right: I’m afraid there’ll come a point when I get told he’s not getting anything out of me anymore — and then what? What’ll happen then? That’s the uncertainty, while my work is also… it’s something for me to hold onto. I’m not saying I want to stop working because that’s simply not possible. I could take early retirement next year but it would cost me a fortune so that’s not going to happen. So I do want to keep working. But you know, if my manager is already talking about seeing whether he’s getting anything out of me, what does that mean? Those are the kinds of statements that make you break out in a sweat and give you sleepless nights. (#6, T1)

#### Moderate versus high persistent level of caregiver burden

Within the group of family caregivers who experienced a persistent level of burden over time, there was variation in the reported level of burden. Some family caregivers experienced a high burden over the course of the illness trajectory of the patient, while others experienced a moderate burden. During the analysis, it became apparent that family caregivers who experienced a higher burden often had a strong sense of responsibility, had high expectations for themselves (Table 2, Q5) and wanted to solve problems themselves. Having less confidence in the ability of others to provide care or believing that care provision is more efficient when they personally provided it, and taking up more caregiving tasks because of this, was also typical for caregivers with a higher burden. These beliefs made it more difficult for them to accept help from others. A very intensive care situation, having little time for relaxation, feeling guilty about moving the care recipient to a nursing home, or feeling guilty about not spending enough time with the care recipient, were features also seen in caregivers with a higher burden. The sudden death of one care recipient while having to provide intensive care to another care recipient and/or experiencing high work pressure was also related to a higher burden (Table 2, Q6). Also, having difficulties with the changing behaviour of the care recipient and experiencing the care situation as psychologically or emotionally tough was linked to a higher burden. The pressure of having to arrange everything, while experiencing difficult communication with municipalities, other organizations and healthcare professionals also increased the burden. In some cases, even though there was flexibility and autonomy at work, high work pressure in combination with a feeling that their supervisor did not understand their situation was related to a higher burden.

Family caregivers who persistently experienced a moderate burden over time often had a positive attitude, shared care tasks with others, and had flexibility and autonomy at work. It is noteworthy that family caregivers who persistently experienced a moderate burden were more likely to have a job in professional healthcare (e.g. a home-care staff member, a nurse, the former CEO of a nursing home). They mentioned that, because of their background in professional healthcare, they had experience with ill people and changing behaviour, they were familiar with the care sector, they knew how and where to arrange help and they had short lines of communication with other healthcare professionals. The caregivers who worked in professional healthcare also felt more comfortable in performing care tasks for their relatives and were sometimes better able to put things in perspective. In some cases, they had worked as a supervisor or CEO, knowing exactly what kinds of arrangements were possible within the organization. However, there were also some family caregivers with a job in healthcare who persistently experienced a high burden. For them, the ‘advantage’ of being a healthcare professional could not compensate for the emotional burden of the situation. In some cases, they found it difficult to focus on their caregiving role as a family member, because they were critical of the work of the healthcare professionals they had to deal with (Table 2, Q7).

### Burden increased over time

In the second group of family caregivers, the burden increased over the course of the illness trajectory. The burden increased when the care became more intensive, when there were acute situations, such as a crisis situation or hospital admission, when the health of the care recipient deteriorated, when the dependence of the care recipient on the family caregiver increased, or when the care recipient was in the terminal phase and death followed (Table 2, Q8). These family caregivers often shared a home with the care recipient. Some caregivers struggled to accept help from others, because it gave them the feeling that they had failed at caregiving (Table 2, Q9). The family caregivers in this group all experienced burnout symptoms over the course of the illness trajectory of their loved one. They reported symptoms such as tiredness, stress, headaches, palpitations, constant adrenaline (Table 2, Q10), or post-traumatic stress disorder (PTSD) from adverse events in the care situation (Table 2, Q11). Some reported that they were in survival mode, felt trapped in the situation or did not have time for their own life. They also experienced a lack of sleep and consistently did not get a good night’s rest (Table 2, Q12). Almost all caregivers in this group were on sick leave from their work at some point in the trajectory because of their burnout symptoms. Not all caregivers received a supportive response at work (Table 2, Q13). The specific role of work in the burden differed between participants. For some caregivers, the workplace and working conditions increased the burden over time, especially when they did not receive understanding and support, had a negative experience with a supervisor or colleague (Table 2, Q14), or were under pressure at work. Some caregivers found the combination of care and paid work stressful as they had the feeling they were falling short in both their work and their caregiving. Self-employment also contributed to a higher burden in some cases. Although self-employment generally comes with high autonomy, these caregivers did not have support options to rely on and it was sometimes difficult to separate work and care time. For others, conditions at work were better, but not sufficient to prevent burnout symptoms when the work pressure was high and the care situation intensive. There was one exception, where the employer gave the family caregiver complete freedom to take time off for a longer period of time without loss of salary or formal arrangements. Although the burden increased due to the intensive care situation, this caregiver did not experience burnout symptoms (#18, Table 1).

### Caring for someone nearing the end of life is emotionally burdensome

Almost all family caregivers found the situation emotionally tough, even when they were satisfied about the way that work and care were arranged (Table 3, Q1). Family caregivers found it hard to see the care recipient’s health deteriorate. They were often very emotional about the transition to a nursing home or hospice when it was no longer possible to keep the care recipient at home. Most caregivers who had lost the care recipient over the course of this study noted that the final weeks or months were most difficult. For a lot of caregivers, it was difficult to estimate how long the situation was going to continue and how the illness would develop. This uncertainty itself was also hard for some caregivers. In the first round of interviews, some caregivers pointed out that the care recipient was very sick and would probably have passed away by the following interview. However, in some cases, the care recipient was still alive at the end of this study, about two years later. Constantly thinking that the care recipient would die soon was emotionally exhausting. They had to adjust expectations, and were often sad or scared. Family caregivers also took the approaching death into account in planning their own lives (Table 3, Q2). They often found it difficult to determine when and how to act when the health of the care recipient declined (Table 3, Q3). This also made it hard to determine when to take compassionate care leave from work. In some cases, caregivers were able to use compassionate care leave or unpaid leave from work for the last weeks of life of the care recipient after good discussions with the professional caregivers about when to take leave (Table 3, Q4). In some cases, family caregivers pointed out that they could cope with the situation because they knew it was not going to last much longer.

**Table 3:** Quotes overarching themes

No	Theme	Quote
Q1	Situation is emotionally tough	The tricky part is simply that the situation is so awful. That’s what actually makes it difficult now; the combination with work is nearly always OK in fact. So I’m pleased with that; in fact that’s one thing I am pleased with. So it’s more that emotional burden. That’s what affects me above all – that’s what makes it tougher now rather than the combination with work. (#17, T2)
Q2	Approaching death of care recipient is emotionally exhausting	Sure, it’s wonderful that [the care recipient lives longer than expected] but it’s also quite draining emotionally. You have all these expectations and then you have to adjust them again and you’re sad again or shocked, and then you’re relieved again. But it’s like that the whole time. And I don’t want to stay away too long at the moment, so I’m always allowing for the fact that it could suddenly get worse next week. […] So I’m thinking: is that serious or not? Right. We have these discussions sometimes with the GP, that he’s terminal now but shouldn’t we stop using that label? But that hasn’t happened so the GP probably does have this expectation that it could end quite quickly. But at the same time he has quite an unusual kind of cancer, so I reckon it must be difficult to predict. Yeah, so it’s both the fact that it costs a lot of time and the fact that it’s emotionally quite draining. (#12, T3)
Q3	Difficult to determine when and how to act	That final period was the toughest. Basically from just before she was admitted to the hospice, in December, until she died. That was really tough. […] I hadn’t had many ups and downs until the end, it was just continuous, the whole time, expecting that there would be more bad news and misery, and then sorting it out. You simply do what’s got to be done. But every time you had a bad news talk, saying that she was so sick, you would have these doubts all the time: should I call the doctor or the hospital now, should I take action or not? Those were tough moments, definitely. So for example, when I could see that she was really weak but she didn’t want me to talk to the oncologist, well, that was difficult. […] But also the whole process of having cancer, that you don’t know when the time will come, the constant uncertainty. That uncertainty is also about you not knowing what you should do. Like OK, I’m really worried but is this now the point where I should actually phone the oncologist: yes or no? And then I’d discuss it with my mother. And that’s very… the fact that you permanently don’t know what the situation is, or what you should do or whether you’re doing the right thing. Right, I think that was actually the hardest part. (#17, T4)
Q4	Hard to determine when to take leave from work	When she was admitted to the hospice, I took short-term care leave. But that was two weeks and when it ended, I went back to work. So I worked two weeks then, and after that I took unpaid leave. So I was pretty much there the whole time then. I coordinated things a bit with the nursing staff there… because I could only get four weeks of long-term leave and I didn’t want it to end just at the point when my mother was getting sicker and close to the end. So I wanted to plan it so that I could use it all for the final days with my mother. And that was what happened; it worked out in the end. I’m pleased that I did that. That I was able to spend that time on her and I didn’t have to worry about my work at the same time, and I didn’t have the stress of combining that. […] It was difficult to estimate. For two years, I thought it wouldn’t be much longer but she kept on going. So at some point, you ask the nurses what their experience tells them is going to happen. You can never give guarantees, but based on your experience how much longer has she got? So right, then I agreed things with them. (#17, T4)
Q5	Death of care recipient also gave some sense of relief	Of course, they too [the daughters] had seen it coming. They were also relieved when she died, I was too — kind of liberated too, because you’ve got rid of that whole illness, of the appointments with the hospital, the stress, not knowing what your day will be like. Crazy things that can happen, and you can do normal stuff again. The three of you can just get in the car and say, how about going to the beach this evening or why don’t we go to McDonald’s, just something crazy. So the normal things are possible again. (#18, T1)
Q6	Return to work difficult because caregiver feels worn out	I spent eight weeks off work after he died. I’m still not back at work fully. I started working again in November. I have to say that my employer was very good about that. I was allowed to do the hours I wanted; if I wanted to work until one o’clock that was fine, if I wanted to work until twelve that was fine, it was all no problem. At the moment, I’m only working until one thirty. I started working half an hour extra in July but I am finding that simply incredibly tiring. So whereas I was able to really keep going during his illness, well, you end up in a kind of void… Yes, it hits you eventually. (#15, T3)
Q7	Grief coach facilitated by employer	I had this sense of being stuck in a quagmire that I couldn’t get out of. So then I went to the practice assistant. I had two appointments with her and she said it was a little bit beyond what she could do. So then I was in contact with a grief coach. […] Well, that isn’t actually covered by the health insurance, which I think is a pretty poor show. But my work decided they would pay for it. They basically felt it was very much in their interests for me to carry on working. (#15, T3)
Q8	In hindsight, caregiver would have accepted help sooner	Start sooner with the neighbourhood care service and home care. Right, in my case, well, you maybe ought to begin when you… um, personally I found it really difficult to ask for help. I had this feeling that I’d failed. Whereas [the care recipient] had actually said a couple of times, “You can’t keep this up and I really don’t mind; it’s OK if you ask them.” But I found it so difficult to take that step of involving the neighbourhood care service because you’re basically handing over to them. And I found that so difficult to do. Looking back, I should have done it a bit earlier. But well, that’s just how it went. You need to be ready to take that step as well. (#15, T3)
Q9	Care situation had affected relationship more than caregiver realized at the time	In 2019, I realized that it was a real emotional and physical burden for me. […] Now, looking back, I can put it into words a bit better. When you’re in the middle of it you just keep going, so there might be something you pick up subconsciously but you don’t do anything with that information. Because I knew anyway that I couldn’t do anything about it. But I’ve talked a lot with my wife over the last while about how I’ve noticed that it’s had a big effect on our relationship. Now we’re working together on kind of coming together again, because we started living separate lives; I think that must have been the case for three or four years. Looking back now, we see… I was spending a lot of time with my mother, but even when I was here, I was really still at my mother’s emotionally. I would plan everything. First, I would go over and care for my mother, then I would see whether I could do anything at home with friends or my wife. As a result, we had a really strange relationship and that’s now a bit… we’re trying to sort of get things back on track again. (#17, T4)
Q10	Lack of aftercare following the care recipient’s death	The aftercare for partners after someone has died. Because at that point… I get it because [the care recipient] was the patient and the ergotherapist visited me once after he died, but the rehabilitation specialist, well, I spoke to them on the phone on the Monday and that was the last time I heard anything. They just abandon you. There’s no aftercare at all. Whereas I do find that very important. So I think that is pretty poor. […] Well, I’m thinking maybe that a grief coach could be added to the rehabilitation team to give that aftercare. Because what you’ve been through is not nothing. Because the grief coach actually said, “If you don’t watch out, you’ll get PTSD”. (#15, T3)
Q11	Role of workplace in burnout	I spoke to various people at my work about my problems. The confidential counsellor, HR, my team manager, the works council. And nobody did anything. That was in 2013 or 2014. It was never dealt with properly. I had various talks. They really made mistakes there. They didn’t give me proper support either in advising me what I could do to make things easier for myself. So they offered to reduce my contract and I’m still suffering the consequences because now I’ve only got a contract for 20 h. A better recommendation would have been to take unpaid leave because now if I get occupational disability it will be based on that figure of 20 h. The occupational specialist also wrote in her report that my employer didn’t do enough. She thinks I’m a ‘medical reducer’, which means that I took measures off my own bat several years ago so that I could carry on working, as I kept cutting my working hours. My employer should have done more to help. (#6, T3)

### Decreasing burden after death

Over the course of this study, the care recipients of seven family caregivers died. For most of these caregivers, the level of burden decreased substantially after the death. Although they were sad, they often also felt relieved because the intensive care demands had stopped and there was room for normal things again (Table 3, Q5). Some caregivers reported that they had been able to prepare for the death of their loved one, because that person had been ill for a longer period of time. Some caregivers felt worn out after the period of caregiving and struggled with feelings of grief and loss. This sometimes complicated their return to work (Table 3, Q6). In most cases, supervisors were supportive and understanding, giving the caregivers space to return to work at their own pace. In some cases, the employer arranged a grief coach to help the family caregiver cope with their grief and loss (Table 3, Q7). Some family caregivers returned to work within a couple of weeks because their work gave them energy and satisfaction. In some cases, caregivers returned to work quickly because they wanted to be loyal to their employer and show their gratitude for the support they had received during the illness trajectory of the care recipient. For some family caregivers, feelings of grief made way for acceptance of the death of their loved one.

### Different view of the trajectory in hindsight

Looking back at the period of caregiving, most caregivers found it hard-going, but were also satisfied with how they had handled the difficult situation. However, during the interviews, it became apparent that family caregivers sometimes had a different view of the trajectory in retrospect compared to when they were actually in the care situation. In earlier interviews, they would, for instance, paint a more optimistic picture compared with when they reflected on the trajectory later on. One caregiver specifically noted that it was easier to realize in hindsight what the situation was really like and how they felt at the time. Also, the more difficult the situation became, the harder it was for them to reflect on how things were going because they went into survival mode. In hindsight, some caregivers wished they had accepted help from others earlier in the trajectory. At the time, it felt like failing to bring in more professional care (Table 3, Q8). In some cases, the family caregiver only realized afterwards that the care situation had negatively affected their relationship with their partner, because they were emotionally unavailable and constantly thinking about the care recipient (Table 3, Q9). Some caregivers noted a lack of aftercare following the care recipient’s death. They felt somewhat abandoned by the healthcare professionals and would have liked more support with their grief (Table 3, Q10).Some caregivers indicated they would have preferred to work fewer hours in hindsight than they actually did in the caregiving period. In some cases, the caregiver indicated that their burnout symptoms could have been avoided if their supervisor had been more supportive in considering changes to their work. Also, caregivers reported that knowing more about the available support options would have helped alleviate pressure (Table 3, Q11). Specific recommendations family caregivers had for other family caregivers, the workplace, healthcare professionals and local authorities are shown in Table 4.

**Table 4:** Advice given by family caregivers to other caregivers, the workplace and local authorities

**Advice for family caregivers in similar position**	
Arranging/accepting help sooner	If people offer help, accept it. In whatever form it comes, even if it’s grocery shopping or “How about I go on a walk with the person who’s sick to give you a bit of a break”. (#10, T3)
Keeping the people around you informed about what is going on and what you need	What I find really important is to keep the people around you — your family and friends — informed so that they know OK, that’s what is going on. And yeah, what I found difficult but others might find easier is accepting help when it’s necessary, so the rest of your immediate family can keep going and find a way to cope. (#14, T3)
Make your boundaries clear	Make your boundaries clear, because I went too far. And do so in good time… but that’s ever so difficult because it’s often an emotional thing, that involvement. It also depends on who you are caring for. Are you caring for your partner or a child or your parents, or are you caring for a neighbour? So I do think… look, when it’s your neighbour it’s easier for you to take a step back than when it’s your parents, for example. That’s the difficult part, because it’s to do with your emotions. (#10, T3)
You are allowed to prioritize yourself/look after yourself	You’re allowed to prioritize yourself, put yourself first. You are the one who has to do it, you have to… you have to cosset yourself. And make sure you stay healthy by doing sports, exercising, looking after yourself. Make a hairdresser’s appointment, do something for yourself. Eat healthily. Then you’ve already made big progress. (#4, T3) Right, always making sure you look after yourself, but that is… Well, I think that everyone probably says that so it’s virtually a useless tip because everyone I speak to says it just happens, as it were. I’d almost be reluctant to give it as a tip because I know… I don’t even know how you could do that. […] I think perhaps it could help if you realized that you… you’re not under any obligation or whatever. You have a duty to keep good care of yourself. If you’re the relative of the person who’s ill, they don’t gain anything either if you run yourself into the ground. Perhaps if you look at it like that, it’ll help you realize that it’s a good idea to look after yourself. (#17, T4)
Get information about the options	Get proper information about the available options. (#7, T3) Make sure you’ve got all the right information. And make sure you have a backup, so that you can share the care with other people, because that really helps. (#1, T3)
Be proactive in arranging care and contacts who could help you	If you know it’s going to be a very long process, you could kind of take that into account at a very early stage. I think that would have helped me. And you could already start looking for people around you who could help you. It’s too late for that once the going gets tough. So you’d already know what contacts you have and what you can do and who you need, so you make plans for things that help. […] Otherwise you’ll always be playing catch-up and be under a lot of stress. There will come a point where you’re playing catch-up because this is a battle you can’t win. But I think anticipating and preparing for things wherever possible could save a lot of stress and worry. (#17, T4)
Trust your instincts	Take charge and stay in charge. And trust your instincts. I think that is more important: feeling OK with things and doing what you’re OK with doing. And if you don’t want to do it every day, even if it’s only twice a week or once a week or once a fortnight, do what you feel is right. There are enough people who say, “I’m not going to go every day”. That’s up to them. (#16, T3)
Make a keepsake box	What I did that could be a tip for other families… Because sometimes you think you’ve got time, but it turns out that time’s far too short. And then you are left with nothing. I was really already working on saying goodbye when he was still there; maybe that wasn’t right, but it does mean I have a lot of memories. I recorded things, and I got photos of the CT scans, you know so that they have something… well, you can tell them Daddy died of cancer when he was only 44, but what does that mean? And I’ve just got all these photos. So they’ll be able to see them, when they’re older too. You can also order a bereavement blanket. Well, that’s up to you, but I got hold of one indirectly. For the children. And it included a very nice letter for the children saying when Daddy’s no longer there and you’re feeling sad, then you can wrap this blanket around you… you know, that kind of thing. You really need that as a family. (#2, T3)
Be open about the care situation at work	Indeed, being open about it at your work too and adopting a leadership role yourself, so not waiting to see what your manager can do for you. But I think you have your own responsibility here and you need to make it something you talk openly about: OK, what am I able to do and what can I really not manage? And I think you have that responsibility towards your colleagues too. What isn’t possible simply isn’t possible, I’m quite convinced about that too, and it’s fine to be honest about that, but you do need to bring it out into the open. In fact, as we were saying just now, take a look at whether you need help, yes or no. And don’t be ashamed to make use of that help. (#14, T3)
Try to see the plus points as well	When you do things, do them attentively, with affection and with dedication, prioritize yourself and the other person too, the person you’re caring for. And try to see the plus points – seize the opportunities. You know, when it’s windy some people will put up a windbreak and others will build a windmill. So that kind of thing. Like when it rains, older people like me will put up an umbrella while kids go and stamp in the puddles. Try to turn it round and see what benefits it has and don’t let yourself be dragged down by a medical… or by a disease, or have your identity reduced to that disease. You’re making the disease into too big a thing then. (#18, T1)
Make contact with fellow sufferers	Share with one another… I’m also on a closed Facebook group for family caregivers… right, sometimes that will give you recognition of what you’re going through or you can let off steam, or just add a comment like, that’s so familiar, good luck with that. Yeah, you help one another a bit. […] Then you feel understood. Then you know these people are in the same situation. So: they understand what it’s like and you understand them too. (#7, T3)
**Advice for supervisors and employers**	
Have a clear policy for informal caregiving rules within the organization	Right, have a clear policy on that. At my work, I see it depends on the team manager. A good example is when I had really big problems with my wife. I needed a few days; I needed time that I essentially no longer had any right to, legally. My team manager called that my ‘personal responsibility’. Then a colleague got news from abroad that his mother who lived abroad had become seriously ill, so he was immediately taken off the roster for two weeks so he could travel abroad and the agreement was that they wouldn’t deduct it from his holiday allowance. That’s not on; you can’t have differences like that. Because then I’m thinking that’s also your personal responsibility. If his mother is ill abroad, then he should just take some holiday leave. (#6, T3)
Point out possible leave schemes to employees	That you encourage the employee to think about what options they have but also point out the possibilities. I looked up those rules myself. But if someone doesn’t take that upon themselves, the manager can suggest it, of course. Say these are the options and what would be most feasible in which situation. (#14, T3)
Help think up solutions	Well, I have to say that in my case, my employer was very good in helping look at the options and what they could do for me. They also found work that I could do from home. They created all that for me, and I found that ever so nice. (#15, T3)
Keep up a more active discussion about the care situation and make agreements	Keep up a more active discussion about it too, I think. If you know someone’s a family caregiver, at some point the person close to them — the manager or team leader or whoever — should ask them, hey, are you still managing to schedule everything? Ask whether anything has changed, or if you made an agreement ask whether that’s still going well. Sit down with that person. Not in the office: go to the cafeteria, reserve some time for the chat, get away from the office environment, go to the restaurant, take a short walk and talk to them like, well I’ve seen this, I see you have that, is there anything I can do for you? How could we explore together what we can do to achieve this, and what can I offer you? (#4, T3)
Make sure the family caregiver feels people are listening	Right, right… well, you’re too busy or… I’ve never reported sick, but how are you going to deal with this? And if you’re not sure what to do, be open about that: I don’t know at the moment what to do for you. It’s not that I’m abandoning you, but I’m not sure at the moment how I can deal with this. Do you want me to carry on with this, or are you saying I should just let it drop? […] Because I think then you feel someone’s listening, even if nothing happens… well, that’s not the right word, but even if your manager says at some point that they don’t have a solution and should we carry on looking or not. At least then someone listened to you. So you have a sense that you’re being taken seriously. (#17, T4)
Tell each other what your expectations are	I think they should have discussions with their own staff. What the staff expect from their manager and whether they could perhaps cut their hours or take temporary care leave. What I mean is, I once had someone who took care leave to look after her father. That was just a couple of weeks but I think managers should be open to it. But well, you do need to see it from both sides and tell one another what your expectations are, because the work still goes on. (#16, T3)
Discuss how the colleagues should be kept informed	I think the communication with the other employees is important. You’ve got to discuss it with the family caregiver too. In this case: right, what should we tell the team and when? So that the caregiver still feels involved in the team and you don’t get a lot of gossip developing and people making all kinds of unnecessary assumptions. I think that’s also an important task for the manager. (#14, T3)
Pay attention to the topic of informal caregiving (and bereavement) in the workplace	What I would like to offer as an idea for them, I think, is that co-workers could pay more attention to their colleague’s grieving process: what it involves and what it means, because I think there’s room for improvement there. Look, they do their best, but I feel it’s quite uncharted territory. […] You’re still sitting there and so people think you can still do all the work you normally did, so everything ends up on your desk again. And nobody looks at how many hours you are working here and whether you can cope with it all, if you are you coping, if they should give someone else some of your tasks. I can work this out with the management, who basically say OK, we’ll take these tasks off you. But you do still find on the work floor that some people aren’t so understanding. (#15, T3)
Give family caregivers time, space, understanding and trust	Pay attention to family caregivers and show your appreciation. Give them space, time and trust. And in particular, don’t push them. Human capital is so much more important for adding value than the other kind of capital, the turnover or… Social capital is so incredibly important: it’s the people who make the difference. The company doesn’t exist at all…It’s just an abstract construction, it’s something that’s put on top. The system doesn’t exist either: we are the system, we are the company. So it’s almost something to do with the culture, how do you deal with… well, how would you personally want to be treated? So right, that’s how you should behave towards someone else. (#18, T1) Be very kind to them and be understanding about the situation. Don’t think “Oh, I’ll just do that”, because that’s not is the way it works. No. And I must say, the arrangements are quite good at my work, fortunately. They’re very flexible. I’m really pleased about that. But I also think there are other employers who really aren’t alert to this and aren’t willing to make the effort either. Sure, that’s the reality you have to deal with in our society today with market forces and everything closing down. Well, it’s simply terrible. (#7, T3) It’s ever so nice when they’re understanding, and to have the space… for the days when you really need to do something with the person you’re caring for, … I think the key thing is being understanding. Well, that and the space… so you can actually give that help. Right, right… I can’t think of anything else, actually. (#12, T4)
**Advice for healthcare professionals**	
Make sure someone gets the same staff and a designated contact in the care team	Right, you know this particular patient is getting care through the Long-Term Care Act so they’re obviously going to need care for a long time. So draw up a roster with a fixed team. But instead, he’s constantly seeing new people. Then they tell you well, we can’t always deliver. I get that, if staff are sick or on leave. But I mean if you… you have ten people, let’s say, who have to deliver the care and you have them available, and you have four appointments with a patient, then you can make sure the same person always visits the patient, at any rate in the morning and the evening. And in the afternoon and when they go to bed… I don’t think that’s particularly difficult. I mean, we draw up rosters here too. So I don’t know what the problem is. But it’s always a different person, you know. Then I reckon, well, if you have long-term patients, you should try to introduce some kind of routine. But that’s not what happens at all. (#8, T3)
More guidance and contact with the family caregiver	Practical tips and, well, the kinds of things you should be looking out for as the partner, if you don’t have home care, because that period could last five months or it could be what we had, nearly a year. Even if you just have some procedure for a phone call once a month, talking to one another, and then an appointment to see the family briefly once every three months. Perhaps you could even have home visits, you know, that’s very much the modern approach. Really a bit more guidance in the whole process, not just looking at the disease side but also more the social side. […] There were a lot of things they [care professionals] didn’t know, where you think well OK, but that happened too. Then there was a phone appointment with the doctor, who never called. There I was, sitting waiting for that call all day. I think that an awful lot of patients and families have experienced that kind of thing. Sure, we had that happen in [hospital] but of course that’s an oncological centre so you think, they really should… that’s the modern approach. Surely you don’t still let people figure things out for themselves? You need guidance. Especially when a family is going to be losing a father or a mother. (#2, T3)
More aftercare and guidance after the patient’s death	The aftercare for partners after someone has died. Because at that point… I get it because [the person with a care need] was the patient and the ergotherapist visited me once after he died, but the rehabilitation specialist, well I spoke to them on the phone on the Monday and that was the last time I heard anything. They just abandon you. There’s no aftercare at all. Whereas I do find that very important. So I think that is pretty poor. […] I’m thinking maybe that a grief coach could be added to the rehabilitation team to give that aftercare. Because what you’ve been through is not nothing. Because the grief coach actually said, “If you don’t watch out, you’ll get PTSD”. There is an awful lot you have to deal with, and well, that really tends to get forgotten. Because I hear this from other people too, that people have difficulty with this. (#15, T3)
Easier procedures for care needs assessments	Well, the reassessment: it’s all still complex and difficult. And something always goes wrong. Then you need to put in a lot of effort to get things straight. You eventually get it sorted, but it just costs an awful lot of energy. Especially assessments for the longer term – rethink the approach. Because now they’re saying: we’ll do it annually because the care situation could change. But you could also delegate that to the person in question, tell them “Let us know if anything changes”. Of course people might keep schtum if the care needs become less and you keep up the care… Well if necessary you could deal with it by contacting the GP, get the GP to draw up an assessment saying the situation is the same, or this has got less or that… You know, just a bit more straightforward. Now you have to go through this whole rigmarole, it costs vast amounts of money and loads of time. Make it all a bit easier. […] You get this huge stack of paperwork staring at you, and a period of uncertainty. It’s so much work that there’s a waiting list for the reassessment and processing it. And you’ve got two different parties because the community nurse does the assessment and then you send it to your health insurer. In our case at any rate. And they too have a backlog. So you keep facing these backlogs. Yup, that’s so frustrating. So then you have to go without money from the personal care budget for two and a half months, well, they simply assume… look, I’m giving care informally so I can say to my wife, “OK, but I’m not going to abandon you. I’ll still help you”. But if you’re using formal care services, they simply won’t turn up if you don’t pay. (#6, T3)
**Advice for local authorities/government bodies**	
Improve communication about the support options and the procedures	I think an awful lot still needs to be done in terms of communication and the lack of clarity especially. I mean it when I say ‘an awful lot’. Look, the basics are all there but it’s not clear to loads of people. Where can they go to, who can they go to, and how seriously are they taken? I think that… that we still have work to do there, even with everyone’s good intentions. Right. So I think that’s the main message in my story, what I come up against. (#11, T2)
Earmark money for supporting family caregivers	To start with, each municipality is allowed to decide for itself how it sets things up. The money the municipalities get isn’t earmarked. So municipality A pumps a lot of that money into youth care, for example, because they’re short of money there. That’s not on. You can’t do that with your own money. If you’re getting money to pay your overheads and you think oh well, it’s quite an expensive month so I’ll use that money for my shopping, your housing corporation will eventually come knocking and say, “You owe us”. That’s not on, it’s weird. […] There are cutbacks but they’re not short of money because no one looks at them because apparently there isn’t a problem, because the informal caregivers just keep going. […] It’s weird that you have money that was meant for a particular purpose and you can channel it somewhere else… you’re really showing contempt for the group it was intended for. These days, informal caregiving is quite a challenge, and then you just take money away from them. That’s a shame but we need it elsewhere, so tough luck. That’s not on. (#6, T3)
No more market forces in the care sector	Well, that’s the reality you have to deal with in society today with market forces and everything closing. Well, it’s simply terrible. No, I would like to make a case for that… well, putting an end to those market forces. The SP [Socialist Party] is also working on that of course. Well, that message needs to be taken on board by the politicians at last. I was really pleased when Hugo de Jonge [Dutch Health Minister] said we’re going to take youth care services off the hands of the municipalities because that is just not working. I thought, hey, they’re making a start. […] The market forces approach has really ruined things. It turns you into a criminal, a fraudster. The authorities have this attitude of “there’s another one who’s trying to get something off us. That’s our money.” That’s how it feels. And guys, you just need people who say… like that complaints lady at [the health insurer], who said, I understand what you’re saying and I’ll sort it out and if there is anything… that gives you breathing space. (#7, T3)

## Discussion

This study aimed to describe the trajectories in the burden of working family caregivers of patients with a life-threatening illness, and to describe which factors in paid work and care were related to changes in the burden over time. In line with prior research [21, 22], almost all family caregivers in this study experienced some level of caregiver burden. Two groups of family caregivers were identified according to their trajectories in the level of burden: those with a persistent level of burden and those with an increasing burden over time. Family caregivers with a persistent level of burden seemed to be at risk for burnout throughout the illness trajectory of their care recipient. When an issue arose, they were often able to cope with the situation by making arrangements in the care or at work. However, they were pushing their limits and often felt exhausted.

Family caregivers with an increasing burden were unable to make sufficient adjustments to alleviate the pressure. For these caregivers, the combination of paid work and family care became too much, resulting in sick leave from work. This is in line with the ‘wear-and-tear’ perspective, which predicts an increase in the burden over time for family caregivers due to the cumulative effects of stressors that exhaust their mental and physical reserves. This might not be surprising, however, since providing care to a loved one nearing the end of life was often emotionally burdensome and intensive. In line with our findings, support for the wear-and-tear perspective has been found in partner caregivers and/or caregivers who live with patients [23, 24].

This study was among the first to describe trajectories of burden among family caregivers based on longitudinal qualitative data. Contrary to previous longitudinal quantitative studies [22, 25], this made it possible to identify events related to changes in burden over time, as well as to describe why these events were of importance. Moreover, opposed to prior research [25], this study did not only focus on the care situation, but also considered the impact of work on care and vice versa. Also, discrepancies between family caregivers’ perceptions during and after the care situation could be identified. The results indicated that the experiences of family caregivers did not only differ between caregivers, but individual experiences also differed over time.

In addition, the framework that has been identified based on the first round of interviews [7] was confirmed by the longitudinal data of the current study. Over time, experiences, feelings and needs regarding family care and the combination with paid work were influenced by four domains (caregiver characteristics, the care situation, the work situation, and the context). These experiences, feelings and needs sometimes had an impact on their health and well-being, or prompted caregivers to take action to improve the situation (e.g. changing jobs or arranging more help with care). Also, changes in health and well-being sometimes had an impact on the situation in multiple domains, for instance, when caregivers experienced an increasing burden and eventually went on sick leave from work.

Our findings correspond with a recent meta-analysis that showed a significant increase in emotional exhaustion when proving care for a relative, which was a risk factor for burnout [26]. Similarly, almost none of the family caregivers in our study experienced a decrease in the burden during the illness trajectory. The burden often only decreased substantially after the care situation had ended. Family caregivers emphasized that they felt sad but relieved after the death of the care recipient, and that there was space for normal things again. Our findings are consistent with other research that has shown that depressive symptoms in bereaved caregivers reduce significantly within three months after death, despite intensive caregiving situations [27]. Prior research has found that, although the burden increased towards the death of the care recipient, about three-quarters of the family caregivers did not perceive this burden as a problem. For these caregivers, providing family care did not feel like a problem because the loved one had previously also cared for them, and providing care felt rewarding [28]. This could possibly help with acceptance and finding closure after the loss of a loved one.

In line with other research [4], family caregivers shared the illness experience of the care recipient, and this often influenced their physical, psychological, social and spiritual well-being. Their needs were dynamic, and varied according to the trajectory [2, 4]. However, the caregiver burden trajectories did not directly reflect the three distinct disease trajectories (namely aggressive cancers, organ failure, frailty or dementia) that have been described by Murray and colleagues [2]. There was no clear difference between the caregiver groups with a persistent or increasing burden in the illness types of their care recipients. In both groups, caregiver burden was mostly related to aspects of the care situation, such as high care intensity, the patient’s increasing dependency, not being able to share care tasks, and acute care situations. Thus, although different illness types impose different requirements on the nature and intensity of care, the degree of dependency of the patient and care availability seemed to be more important in determining the burden and specific support needs. Similarly, fatigue and the level of dependency on the family caregiver have been found in prior research to be associated with caregiver burden and depressive symptoms [29, 30].

Demanding care situations made it difficult to combine care with paid work, especially in jobs with little understanding from colleagues/supervisors or few support options. This corresponds with other studies that have shown that family caregivers who combine work and care experienced the highest burden, and that higher patient dependency in combination with employment was related to caregiver stress [6, 21]. Among the groups of caregivers with a persistent or increasing burden, there were two cases of caregivers whose course of burden was slightly different compared to the rest in the group. Their cases showed that burnout symptoms and long-term sick leave from work could be prevented if there is understanding, flexibility, freedom to take time off from work without loss of salary or formal arrangements, or by making substantial changes in work (e.g. changing to a job with more flexibility and less demanding tasks). In agreement with a scoping review that showed that flexible work arrangements were essential for caregivers [14], our findings also suggest that work adjustments and support at work could make a difference in preventing burnout and sick leave among working family caregivers. Similarly, the results showed that caregivers who received support and had flexibility in their job also returned to work earlier after the care recipient’s death. Returning to a supportive work environment could help employees pick up their work again [31]. This emphasizes that giving family caregivers help in combining paid work with care tasks could benefit both the family caregiver and the employer in the long term.

Most of the family caregivers who persistently experienced a moderate burden had a job in professional healthcare. Having professional experience with caregiving and being familiar with the care sector might make family caregiving easier for these caregivers. This is in line with the earlier finding that self-efficacy for coping with the patient’s illness was negatively related to caregiver burden [22]. However, prior studies have also indicated that “double-duty caregivers” (i.e. professional caregivers with family caregiving tasks) are at risk of developing symptoms of overload and often experience mental and physical pressure [32, 33]. Concomitantly, double-duty caregivers often have high expectations for themselves and want to solve problems themselves because they know how to perform care tasks [32]. Flexibility and understanding from the workplace, as well as discussing the care situation with their supervisor, are important in identifying issues and allowing customized support to be offered [32].

### Methodological considerations

The longitudinal design, in which 53 interviews were carried out with 17 participants over a time period of 2.5 years, provided rich and detailed information. Moreover, this made it possible to further clarify themes regarding the combination of paid work and family care at the end of life that were discussed in prior interviews. This approach also allowed differences to be identified in the views on how the combination of paid work and family care was arranged at various time points in the illness trajectory. Interviewing the same participants multiple times also builds trust between the participant and the interviewer, and this enhanced rapport might have made it easier to discuss difficult topics. Moreover, this helped us to interview family caregivers who were sometimes under extreme pressure or even on sick leave from work, and learn about their experiences. It could be that these people would not have participated in such research at that time if they had not already been familiar with it. In this way, we were able to tap into a wide range of experiences. At the same time, some of these caregivers emphasized that they valued being able to share their stories and be listened to.

In addition, analysing multiple interviews from the same participant as a single unit made it possible to create individual timelines of changes in burden, work and care. Although the course of burden over time was roughly the same for the caregivers within each group, experiences with caregiving were heterogeneous and changed over time [24]. Some family caregivers, however, did not have a particularly clear course of burden. Given that their experiences were dependent on various aspects of the care and work situation within a certain time period, we should refrain from ‘labelling’ individual family caregivers on the basis of the burden trajectories. In addition, not all family caregivers were in the same phase of the illness trajectory during the interviews. It has been found that the burden increases particularly as the patient’s illness progresses and in the terminal stage [28, 34]. Hence, it could be that for some of the caregivers who experienced persistent levels of burden during this study, the burden did eventually increase (or decrease) closer to the death of the care recipient. However, this would likely not have led to different trajectories at the group level, since all burden trajectories included family caregivers who had lost the care recipient during the study period. Future research could take the duration and phase of the illness trajectory into account when investigating burden trajectories of family caregivers of a patient at the end of life.

Family caregivers were recruited and included via general practitioners. This could be considered as a strong point, since the general practitioners had detailed information about patients with a life-threatening illness and were often in contact with the family caregivers as well. However, there is a possibility that general practitioners refrained from asking family caregivers in intensive care situations. Even so, we did not miss out on this group since some family caregivers in intensive care situations applied to take part in the study in response to the posters that were placed in a hospital. Furthermore, although we sought for maximum variation in the sample in terms of gender, working hours, sector, illness type and intensity of care, the views and experiences of family caregivers with certain characteristics might be underrepresented. This could for instance be the case for male caregivers or family caregivers with a non-Western cultural background. Prior research has indicated that male caregivers report less of a burden compared to female caregivers, and that gender differences in the caregiver’s burden increase over time among family caregivers who provide end-of-life care in the home setting [30]. Furthermore, earlier studies have found that cultural norms and values among family caregivers with a non-Western cultural background might also influence their caregiving experiences [35, 36]. Hence, it could be that these caregivers have different experiences with the combination of paid work and family care at the end of life, and, accordingly, have a different burden trajectory. Future research could give more emphasis to gender and cultural norms in distinguishing caregiver burden trajectories in the combination of paid work and family care at the end of life.

### Practical implications

The family caregivers’ perceptions of the situation often changed over the course of the study. Some caregivers reported in earlier interviews that they had good arrangements for the combination of paid work and family care, while in later interviews they said that this was not the case (or not anymore) and it had been quite tough when they looked back at the situation at that point. This suggests that some family caregivers may find it difficult to recognize or identify unmet support needs at the time when the need is most urgent. It could also be the case that, for some caregivers, not acknowledging that the situation is too demanding might function as a coping mechanism allowing them to keep going. This could be problematic, especially if they refrain from seeking or accepting help and support while burnout is looming.

In agreement with earlier findings [4], key time points when family caregivers might have unmet support needs are the diagnosis, transitions in care (e.g. hospital admission, returning home after a hospital stay, and transition to a nursing home or hospice), when a disease-modifying or potentially curative treatment is stopped, in the terminal stage and after death. Healthcare professionals, such as nurses and home-care staff, could play an important role in supporting the family caregivers of a patient at the end of life at these times. They could use needs assessment tools to discuss potential unmet support needs. The Carer Needs Assessment Tool (CSNAT), for example, has proved to be an effective tool to do this [37, 38]. Moreover, given that the perceptions of family caregivers about how things were arranged often differed over time, discussing potential unmet support needs early on and at key time points in the illness trajectory might prevent a situation in which caregivers keep on going and providing care themselves, while there could be other support options available that they are unaware of or that did not cross their minds. In addition, the results showed that providing care to multiple persons contributed to burden. Given that in the Netherlands, about one in three family caregivers provide care to two or more persons and that this number is likely to further increase in the near future [39, 40], healthcare professionals could be more alert to unmet support needs among family caregivers with care tasks for multiple care recipients.

A supportive workplace appeared to be important in helping family caregivers combine care with work responsibilities. It is vitally important to discuss the theme of combining paid work with family caregiving by creating a safe environment for employees to share worries or issues that complicate the combination of paid work and family care for them. Supervisors could try to accommodate the needs of the caregiving employee by discussing possible solutions and offering tailored support, for instance in the form of flexible work hours, different tasks or working remotely. In addition, organizations should communicate policies regarding formal arrangements more actively, since not all caregivers were aware what support options were on offer. Social workers and occupational physicians could play an essential role in the provision of support for grieving employees and promoting awareness among supervisors [31].

## Conclusion

This study showed that it was very common for family caregivers who combine paid work and care at the end of life to experience a care burden. Providing care to a loved one nearing the end of life was often emotionally burdensome and intensive. The burden did not decrease over time and was mostly related to aspects of the care situation. Although the experiences with the combination of paid work and family care at the end of life were heterogeneous and differed over time, two groups were identified in which family caregivers had a similar course in the level of burden. More specifically, one group of family caregivers experienced a persistent level of burden over time, while family caregivers in the other group experienced an increasing burden. The latter group was unable to make sufficient adjustments, which often resulted in burnout symptoms and sick leave from work. To facilitate the combination of paid work and family care, and reduce the risk of burnout, more support is needed from employers and healthcare professionals during the illness trajectory of the patient and after death. Bereaved family caregivers also warrant more attention from their supervisors and occupational physicians in order to facilitate their return to work.

## Supplementary Information

**Additional file 1. **COREQ checklist. Consolidated criteria for reporting qualitative research (COREQ): 32-item checklist.

**Additional file 2. **Criteria for methodological rigor. Criteria for methodological rigor in qualitative studies and applied strategies.

## Data Availability

The data that support the findings of this study are available from Amsterdam UMC but restrictions apply to the availability of these data, which were used under license for the current study, and so are not publicly available. Data are however available from the authors (hrw.pasman@amsterdamumc.nl) upon reasonable request and with permission of Amsterdam UMC.

## Abbreviations

CEO: Chief executive officer
COREQ: Consolidated criteria guidelines for reporting qualitative research
CSNAT: Carer Needs Assessment Tool
FB: Author Femmy Bijnsdorp
HRP: Author H Roeline Pasman
BP: Author Bregje Onwuteaka-Philipsen
FMCG: Fast Moving Consumer Goods

## References

[1] Martín JM, Olano-Lizarraga M, Saracíbar-Razquin M (2016). The experience of family caregivers caring for a terminal patient at home: A research review. Int J Nurs Stud.

[2] Murray SA, Kendall M, Boyd K, Sheikh A (2005). Illness trajectories and palliative care. Bmj.

[3] Andersson MA, Monin JK (2018). Informal care networks in the context of multimorbidity: Size, composition, and associations with recipient psychological well-being. J Aging Health.

[4] Murray SA, Kendall M, Boyd K, Grant L, Highet G, Sheikh A (2010). Archetypal trajectories of social, psychological, and spiritual wellbeing and distress in family care givers of patients with lung cancer: secondary analysis of serial qualitative interviews. BMJ.

[5] Bijnsdorp FM, Van Der Beek AJ, Pasman HRW, Boot CR, De Boer AH, Plaisier I (2019). Home care for terminally ill patients: the experiences of family caregivers with and without paid work. BMJ Support Palliat Care..

[6] Lindt N, van Berkel J, Mulder BC (2020). Determinants of overburdening among informal carers: a systematic review. BMC Geriatr.

[7] Bijnsdorp FM, Onwuteaka-Philipsen BD, Boot CRL, Beek AJvd, Klop HT, Pasman HRW. Combining paid work and family care for a patient at the end of life at home: insights from a qualitative study among caregivers in the Netherlands. BMC Palliat Care. 2021;20(93):1-13. 10.1186/s12904-021-00780-9 PMC8228921 34167518

[8] Oldenkamp M, Bültmann U, Wittek RP, Stolk RP, Hagedoorn M, Smidt N (2018). Combining informal care and paid work: The use of work arrangements by working adult-child caregivers in the Netherlands. Health Soc Care Community.

[9] Wadhwa D, Burman D, Swami N, Rodin G, Lo C, Zimmermann C (2013). Quality of life and mental health in caregivers of outpatients with advanced cancer. Psychooncology.

[10] Schulz R, Czaja S, Sharit J, James J (2020). The Intersection of Family Caregiving and Work: Labor Force Participation, Productivity, and Caregiver Well-Being. Current and Emerging Trends in Aging and Work.

[11] Vuksan M, Williams A, Crooks V (2012). Family friendly policies: accommodating end-of-life caregivers in workplaces. Int J Workplace Health Manag.

[12] Park SM, Kim YJ, Kim S, Choi JS, Lim H-Y, Choi YS (2010). Impact of caregivers’ unmet needs for supportive care on quality of terminal cancer care delivered and caregiver’s workforce performance. Support Care Cancer.

[13] Vuksan M, Williams AM, Crooks VA (2012). The workplace perspective on supporting family caregivers at end of life: evaluating a new Canadian social program. Community Work Fam.

[14] Spann A, Vicente J, Allard C, Hawley M, Spreeuwenberg M, de Witte L (2019). Challenges of combining work and unpaid care, and solutions: A scoping review. Health Soc Care Community.

[15] Tong A, Sainsbury P, Craig J (2007). Consolidated criteria for reporting qualitative research (COREQ): a 32-item checklist for interviews and focus groups. Int J Qual Health Care.

[16] Lincoln YS, Guba EG (1986). But is it rigorous? Trustworthiness and authenticity in naturalistic evaluation. Evaluation Policy.

[17] Thomas E, Magilvy JK (2011). Qualitative rigor or research validity in qualitative research. J Spec Pediatr Nurs.

[18] Murray SA, Kendall M, Carduff E, Worth A, Harris FM, Lloyd A (2009). Use of serial qualitative interviews to understand patients’ evolving experiences and needs. Br Med J.

[19] Braun V, Clarke V (2006). Using thematic analysis in psychology. Qual Res Psychol.

[20] Strauss A, Corbin J (1998). Basics of qualitative research techniques.

[21] Hsu T, Loscalzo M, Ramani R, Forman S, Popplewell L, Clark K (2014). Factors associated with high burden in caregivers of older adults with cancer. Cancer.

[22] Lee YH, Liao YC, Shun SC, Lin KC, Liao WY, Chang PH (2018). Trajectories of caregiver burden and related factors in family caregivers of patients with lung cancer. Psychooncology.

[23] Swinkels JC, Broese van Groenou MI, de Boer A, van Tilburg TG. Male and Female Partner-Caregivers’ Burden: Does It Get Worse Over Time? The Gerontologist. 2018. 10.1093/geront/gny132 30321338

[24] Ornstein K, Gaugler JE, Zahodne L, Stern Y (2014). The heterogeneous course of depressive symptoms for the dementia caregiver. Int J Aging Hum Dev.

[25] Götze H, Brähler E, Gansera L, Schnabel A, Gottschalk-Fleischer A, Köhler N (2018). Anxiety, depression and quality of life in family caregivers of palliative cancer patients during home care and after the patient’s death. Eur J Cancer Care.

[26] Gérain P, Zech E (2021). Do informal caregivers experience more burnout? A meta-analytic study. Psychol Health Med.

[27] Schulz R, Mendelsohn AB, Haley WE, Mahoney D, Allen RS, Zhang S (2003). End-of-life care and the effects of bereavement on family caregivers of persons with dementia. NEJM.

[28] De Korte-Verhoef MC, Pasman HRW, Schweitzer BP, Francke AL, Onwuteaka-Philipsen BD, Deliens L (2014). Burden for family carers at the end of life; a mixed-method study of the perspectives of family carers and GPs. BMC Palliat Care.

[29] Jin I, Tang D, Gengaroli J, Perry KN, Burlutsky G, Craig A (2021). Cross-sectional study evaluating burden and depressive symptoms in family carers of persons with age-related macular degeneration in Australia. BMJ Open.

[30] Guerriere D, Husain A, Zagorski B, Marshall D, Seow H, Brazil K (2016). Predictors of caregiver burden across the home-based palliative care trajectory in Ontario. C anada Health Soc Care Community.

[31] Wilson DM, Rodríguez-Prat A, Low G (2020). The potential impact of bereavement grief on workers, work, careers, and the workplace. Soc Work Health Care.

[32] Detaille SI, De Lange A, Engels J, Pijnappels M, Hutting N, Osagie E (2020). Supporting Double Duty Caregiving and Good Employment Practices in Health Care Within an Aging Society. Front Psychol.

[33] Boumans NP, Dorant E (2014). Double-duty caregivers: healthcare professionals juggling employment and informal caregiving. A survey on personal health and work experiences. J Adv Nurs..

[34] Grunfeld E, Coyle D, Whelan T, Clinch J, Reyno L, Earle CC (2004). Family caregiver burden: results of a longitudinal study of breast cancer patients and their principal caregivers. CMAJ.

[35] Pharr JR, Dodge Francis C, Terry C, Clark MC (2014). Culture, caregiving, and health: Exploring the influence of culture on family caregiver experiences. Int Sch Res Notices.

[36] Gérain P, Zech E (2019). Informal caregiver burnout? Development of a theoretical framework to understand the impact of caregiving. Front Psychol.

[37] Grande GE, Austin L, Ewing G, O’Leary N, Roberts C (2017). Assessing the impact of a Carer Support Needs Assessment Tool (CSNAT) intervention in palliative home care: a stepped wedge cluster trial. BMJ Support Palliat Care.

[38] Diffin J, Ewing G, Harvey G, Grande G (2018). Facilitating successful implementation of a person-centred intervention to support family carers within palliative care: a qualitative study of the Carer Support Needs Assessment Tool (CSNAT) intervention. BMC Palliat Care.

[39] Boer Ad, Klerk, M de, Verbeek-Oudijk, D., Plaisier, I. Blijvende bron van zorg. The Hague: The Netherlands Institute for Social Research 2020.

[40] Kooiker S, de Jong A, Verbeek-Oudijk D, de Boer A. Toekomstverkenning mantelzorg aan ouderen in 2040. The Hague: The Netherlands Institute for Social Research; 2019.

