# Supplementary material for: Caregiver’s burden at the end of life of their loved one: insights from a longitudinal qualitative study among working family caregivers
Source: BMC Palliat Care. 2022 Aug 10;21:142. doi: 10.1186/s12904-022-01031-1 (PMC9364551; doi:10.1186/s12904-022-01031-1)
Supplement: Supplementary file 1 — Additional file 1. COREQ checklist. Consolidated criteria for reporting qualitative research (COREQ): 32-item checklist. [file 12904_2022_1031_MOESM1_ESM.docx]

**Additional file 1 – Consolidated criteria for reporting qualitative studies (COREQ): 32-item checklist**

| No | Item | Guide questions/description | Section and page number in manuscript |
| --- | --- | --- | --- |
| Domain 1: Research team and reflexivity |  |  |  |
| Personal Characteristics |  |  |  |
| 1. | Interviewer/facilitator | Which author/s conducted the interview or focus group? | Methods, data collection, page 5 |
| 2. | Credentials | What were the researcher's credentials? E.g. PhD, MD | The primary researcher holds a master’s degree in Sociology, a master’s degree in Communication Science and a master’s degree in Epidemiology, additional file 1. |
| 3. | Occupation | What was their occupation at the time of the study? | The primary researcher is a PhD student, additional file 1 |
| 4. | Gender | Was the researcher male or female? | Methods, data collection, page 5 |
| 5. | Experience and training | What experience or training did the researcher have? | Methods, data collection, page 5 |
| Relationship with participants |  |  |  |
| 6. | Relationship established | Was a relationship established prior to study commencement? | There was no relationship established prior to the study. Participants were recruited via general practitioners, posters in a Dutch academic hospital and an item in the corporate newsletter of this hospital. In subsequent interviews, the participants were familiar with the researcher from the first interview. Methods, Design and participants, page 5. |
| 7. | Participant knowledge of the interviewer | What did the participants know about the researcher? e.g. personal goals, reasons for doing the research | Participants were informed about the reasons for doing this study in the information letter. They were not informed about researchers personal goals, additional file 1. |
| 8. | Interviewer characteristics | What characteristics were reported about the interviewer/facilitator? e.g. Bias, assumptions, reasons and interests in the research topic | There were no characteristics of the researcher to report that might have led to bias or assumptions, additional file 1. |
| Domain 2: study design |  |  |  |
| Theoretical framework |  |  |  |
| 9. | Methodological orientation and Theory | What methodological orientation was stated to underpin the study? e.g. grounded theory, discourse analysis, ethnography, phenomenology, content analysis | Methods, Data analysis, page 6 |
| Participant selection |  |  |  |
| 10. | Sampling | How were participants selected? e.g. purposive, convenience, consecutive, snowball | Methods, Design and participants, page 4-5 |
| 11. | Method of approach | How were participants approached? e.g. face-to-face, telephone, mail, email | Methods, Design and participants, page 4-5 |
| 12. | Sample size | How many participants were in the study? | Methods, Data collection, page 5 |
| 13. | Non-participation | How many people refused to participate or dropped out? Reasons? | Methods, Data collection, page 5 |
| Setting |  |  |  |
| 14. | Setting of data collection | Where was the data collected? e.g. home, clinic, workplace | Methods, Data collection, page 5 |
| 15. | Presence of non-participants | Was anyone else present besides the participants and researchers? | There were no non-participants present at the interviews, additional file 1. |
| 16. | Description of sample | What are the important characteristics of the sample? e.g. demographic data, date | Table 1, Characteristics of participants, page 5 |
| Data collection |  |  |  |
| 17. | Interview guide | Were questions, prompts, guides provided by the authors? Was it pilot tested? | Methods, Data collection, page 5 |
| 18. | Repeat interviews | Were repeat interviews carried out? If yes, how many? | Methods, Data collection, page 5 |
| 19. | Audio/visual recording | Did the research use audio or visual recording to collect the data? | Methods, Data collection, page 5 |
| 20. | Field notes | Were field notes made during and/or after the interview or focus group? | Methods, Data collection, page 5 |
| 21. | Duration | What was the duration of the interviews or focus group? | Methods, Data collection, page 5 |
| 22. | Data saturation | Was data saturation discussed? | Only in the first paper of this longitudinal study; in the current paper we have followed the family caregivers over time and described their burden trajectories. |
| 23. | Transcripts returned | Were transcripts returned to participants for comment and/or correction? | The interpretations of the researcher were checked by the participant after summarizing the most important themes during and at the end of the interview. The interviews were audio-recorded and transcribed verbatim. Methods, Data collection, page 5 |
| Domain 3: analysis and findings |  |  |  |
| Data analysis |  |  |  |
| 24. | Number of data coders | How many data coders coded the data? | Methods, Data analysis, page 6 |
| 25. | Description of the coding tree | Did authors provide a description of the coding tree? | Methods, Data analysis, page 6 |
| 26. | Derivation of themes | Were themes identified in advance or derived from the data? | Methods, Data analysis, page 6 |
| 27. | Software | What software, if applicable, was used to manage the data? | Methods, Data analysis, page 6 |
| 28. | Participant checking | Did participants provide feedback on the findings? | A summary of the results was returned to the participants |
| Reporting |  |  |  |
| 29. | Quotations presented | Were participant quotations presented to illustrate the themes / findings? Was each quotation identified? e.g. participant number | Table 2/3, Quotes |
| 30. | Data and findings consistent | Was there consistency between the data presented and the findings? | To the authors knowledge there is consistency between the data presented and the findings |
| 31. | Clarity of major themes | Were major themes clearly presented in the findings? | To the authors knowledge the major themes were clearly presented in the findings |
| 32. | Clarity of minor themes | Is there a description of diverse cases or discussion of minor themes? | To the authors knowledge minor themes or diverse cases are discussed within the results section |
