# Supplementary material for: Caregiver’s burden at the end of life of their loved one: insights from a longitudinal qualitative study among working family caregivers
Source: BMC Palliat Care. 2022 Aug 10;21:142. doi: 10.1186/s12904-022-01031-1 (PMC9364551; doi:10.1186/s12904-022-01031-1)
Supplement: Supplementary file 2 — Additional file 2. Criteria for methodological rigor. Criteria for methodological rigor in qualitative studies and applied strategies. [file 12904_2022_1031_MOESM2_ESM.docx]

**Additional file 2 – Criteria for methodological rigor in qualitative studies and applied strategies**

| **Rigor criteria (1, 2)** | **Purpose / meaning** | **Applied strategies** |
| --- | --- | --- |
| Credibility | To allow others to recognize the experiences described within the study through the interpretation of participants' experiences. | - Member checking and prolonged time spent with the participants: The interpretations of the researcher were checked by the participant after summarizing the most important themes during and at the end of the interview. In addition, some of the themes were further discussed in subsequent interviews to ensure that the interpretations of the researcher were recognized by the participants as accurate representations of their experiences. Additionally, all participants received a summary of the results of the study (i.e. a description of the trajectories). - Using words of the participants: In describing the trajectories, we stayed as close as possible to the words used by the participants. In addition, a substantial amount of quotes was used to illustrate (changes in) the trajectories and overarching themes. |
| Transferability | The ability to transfer research findings or methods from one group to another. | - A detailed description of the demographical characteristics the study population and the geographic boundaries of the study was provided (i.e. the Netherlands). - In the methodological considerations certain subgroups of family caregivers were discussed on which the trajectories may or may not apply. |
| Dependability | When another researcher can follow the decision trail used by the researcher. | - A detailed description of the research process, including the purpose of this study, how participants were selected, how data was collected, how long data collection lasted, which steps were taken in the analytical process and how the trajectories were created and the interpretation of the trajectories were provided. |
| Confirmability | Refers to extend the confidence that the results would be confirmed or corroborated by other researchers. | - The researcher made field notes regarding insights directly after the interviews. In addition, an effort was made to go into the interviews with an open mind and to follow the direction of the participants in the conversations through further inquiry by probing questions. - Diversity in the expertise and backgrounds of the involved researchers contributed to the confirmability of the study results. |

1. Lincoln YS, Guba EG. But is it rigorous? Trustworthiness and authenticity in naturalistic evaluation. Evaluation Policy. 1986;1986(30):73-84.

2. Thomas E, Magilvy JK. Qualitative rigor or research validity in qualitative research. J Spec Pediatr Nurs. 2011;16(2):151-5.
